# Memory recall in arousing situations – an emotional von Restorff effect?

**DOI:** 10.1186/1471-2202-7-57

**Published:** 2006-07-24

**Authors:** Daniel Wiswede, Jascha Rüsseler, Simone Hasselbach, Thomas F Münte

**Affiliations:** 1 Department of Psychology II, Neuropsychology Unit, Otto-von-Guericke University Magdeburg, Germany

## Abstract

**Background:**

Previous research has demonstrated a relationship between memory recall and P300 amplitude in list learning tasks, but the variables mediating this P300-recall relationship are not well understood. In the present study, subjects were required to recall items from lists consisting of 12 words, which were presented in front of pictures taken from the IAPS collection. One word per list is made distinct either by font color or by a highly arousing background IAPS picture. This isolation procedure was first used by von Restorff. Brain potentials were recorded during list presentation.

**Results:**

Recall performance was enhanced for color but not for emotional isolates. Event-related brain potentials (ERP) showed a more positive P300-component for recalled non-isolated words and color-isolated words, compared to the respective non-remembered words, but not for words isolated by arousing background.

**Conclusion:**

Our findings indicate that it is crucial to take emotional mediator variables into account, when using the P300 to predict later recall. Highly arousing environments might force the cognitive system to interrupt rehearsal processes in working memory, which might benefit transfer into other, more stable memory systems. The impact of attention-capturing properties of arousing background stimuli is also discussed.

## Background

Distinctiveness affects memory performance. Subjects are better in recalling items from a list that are distinct in at least one dimension. This phenomenon is called the von-Restorff or VR-effect [1]. Distinctiveness of an item can be created by changing the color, the size, the meaningfulness, the background color or many other aspects of a stimulus [see [2] for techniques to isolate items]. As an example, Fabiani & Donchin [3] visually presented lists of 20 words which included physical (different font size) or semantic isolates (different semantic category). Memory for the isolated items was better than for standard items in free recall as well as recognition tests.

There are different approaches to explain the superior memory performance for isolated items. According to the total-time hypothesis, isolated items are rehearsed for a longer time in working memory, leading to a relative increase in rehearsal compared to non-isolated items [4]. Alternatively, subjects could consider isolated items as a special category in a free recall task [3,5]. This is supported by the finding that the reproduction of isolated items tends to be organized: In the word list produced in the recall phase, subjects recall physically isolated items more often at the end, whereas they produce semantic isolates more often at the beginning [3].

The current study was conducted to further delineate the relationship between item distinctiveness and recall performance by introducing arousing or physical attributes to the words to be learned. In particular, the experiment aimed at the description of the neural correlates of distinctiveness effects through the recording of event-related brain potentials (ERPs). To put the observed brain potential modulations into perspective, we will provide a short review on how distinctiveness, memory performance and emotional information affect ERP components.

### Novelty

Research has shown that novel (as well as isolated, distinct) events trigger a variety of neural processes which are related to attention, perception, learning and memory. Stimulus novelty can be examined by comparing the behavioral and neural responses elicited by the first and repeated presentation of a stimulus. However, the present paper focuses on another form of distinctiveness, namely contextual novelty, which is given when a stimulus occurs in an unexpected context [6].

In ERPs, novel or distinct stimuli elicit an increased positive waveform between 250 and 400 ms [7]. This ERP component is regarded as a member of the "P300 family" and labeled novelty P3 or P3a [6,8]. The P3a is not tied to any particular modality, it can be elicited even when a stimulus is not task-relevant or if it is ignored [6]. It has been argued that the P3a reflects mechanisms engaged in preparing the organism for speeded responses to biologically significant stimulus changes.

Event-related fMRI studies showed that contextually novel stimuli elicited increased BOLD responses in the ventrolateral prefrontal cortex, the insula, and the cingulate gyrus [see [6] for overview]. Strange and colleagues [9] postulated a generic "deviance detection system", which is activated by different kinds of deviance attributes (semantic, perceptual or emotional deviants). It involves right inferior prefrontal and bilateral posterior fusiform cortex. Most importantly, it has been shown that contextually novel events elicit initial activation in the hippocampus, which links processing of novel stimuli to structures directly involved in generating memory [10].

### Difference due to subsequent memory (DM-effect)

ERPs have also been used to predict later recall in list learning tasks. A consisting finding has been a greater positive ERP deflection during encoding for those items that are subsequently recalled compared to items that cannot be recalled. This effect has been labeled "difference due to subsequent memory" or DM-effect [11].

The DM effect has been found in a variety of experimental settings [see [12], for overview]: for nouns in free recall or recognition tasks [3,11,13], for high and low frequency words [14], for physically and semantically isolated words [3], and for picture stimuli in a recognition task [15]. Some researchers have explained this effect within a P300 framework. However, the DM-effect [[11], see [12], for overview] extends over several hundred milliseconds and, thus, has a different timing than the P300 component. Friedman, Ritter and Snodgrass [16] suggested that the DM effect "... could be a unitary ERP activity with a unique functional role that overlaps several ERP deflections, or it could reflect the contribution of several overlapping components, each reflecting a different process."

Functional imaging studies have repeatedly shown the involvement of the inferior frontal cortex and the medial temporal lobe (MTL) including the hippocampus in the generation of the DM-effect [12,17]. Importantly, the same prefrontal, medial temporal and lateral temporal regions (i.e. hippocampus) that generate the DM-effect are also active during detection of stimulus novelty [18].

### Novelty and memory

To detect novelty, it is required to compare incoming information with content stored in memory. Therefore, it is reasonable to assume that structures involved in novelty detection are also crucial in encoding and retrieval. Recent work supports this idea: Contextually novel events elicit initial activation in the hippocampus, a brain structure directly involved in encoding and recall of memory content [10,19]. Lisman and Grace [19] proposed a functional loop comprising the hippocampus and the ventral tegmental area, linking both, novelty detection and memory. The hippocampus detects rapidly incoming information that is not stored in long term memory. Then it generates a novelty signal, which is conveyed through various structures (subiculum, nucleus accumbens) to the ventral tegmental area. The upward arm of this loop increases dopamine in the hippocampus, which facilitates long-term potentiation and learning.

### ERPs and the von-Restorff effect

If a stimulus differs from other stimuli in a series by a distinct feature, this feature has to be encoded in working memory in addition to the item-specific semantic information. According to the context updating hypothesis of the P300 [20], additional encoding should be reflected in an increased P300 amplitude. In an early study [21], subjects were presented with series of 20 words that had to be recalled after every series. Most of the word lists contained a distinct word (different font size). An increased P300 and better memory performance for the isolated items was observed for subjects that reported using primarily a rote rehearsal strategy (based on silent repetition of the word list). In contrast, subjects using an elaborative strategy (making up sentences or stories) did not show increased performance for the isolated words and no P300-recall-relationship, but an increased overall recall rate. Instead, they showed a 'frontal positive slow wave', which was enhanced for subsequently recalled items [21]. In a subsequent study, subjects were instructed to use an elaborative strategy in one, and a rote strategy in the other of two recording sessions. A larger P300 was found for recalled compared to non-recalled items in the rote rehearsal strategy [22]. This effect was seen for the isolated as well as for all other words. However, no differences in the P300 between recalled and non-recalled items were found for the elaborative strategy. Instead, a 'frontal positive slow wave' starting at 800 ms was more pronounced for the elaborative strategy, with subsequently recalled words being more positive. Apparently, different encoding strategies are reflected in separate ERP components [see also [23]].

In the studies discussed above, distinctiveness could not be separated from the item that had to be learned, the distinct information was "integral". Integral dimensions cannot be separated from the stimulus to be encoded because they (a) define the relevant aspects of the items or (b) occupy the same spatial location [23]. Even when the semantic category is changed, the distinctiveness attribute is still integral to the word. In addition to an integral isolate word (increased font size), Otten and Donchin [23] used frames at a close or at a far distance to make a word distinct in a non-integral manner. For non-isolated items, they found a larger P300 for recalled than for non-recalled items. Changing the size of the word (integral attribute change) also resulted in an increased P300 for subsequently recalled items. In the non-integral condition (isolated by a frame in far distance), the P300 was smaller than in the integral condition and there was no relationship between P300 amplitude and subsequent recall. Instead, a larger frontal slow wave was seen for subsequently recalled framed words (non-integral isolates). Free recall performance was increased for isolated items, regardless of whether distinctiveness was integral or not. Those findings imply that it is crucial how distinctiveness is created. Integral distinctiveness attributes had to be fully processed because "processing the size of the characters may be a necessary step towards processing the orthographical, phonological and/or semantic attributes relevant for memorization" [23]. This resulted in more context updating and a larger P300 component. Other organizational processes might be reflected in the frontal positive slow wave, since the recall performance was also increased for non-integral distinct items. The context updating hypothesis thus might be suitable for explaining the standard DM effect for integral isolates, but not for the frontal DM-effect seen for non-integral items.

### Memory and emotions

It is well-established that memory performance is influenced markedly by emotions. One way to classify emotions is to use a two-dimensional affective space, with one axis describing valence (ranging from unpleasant to pleasant) and the other axis describing arousal (ranging from calm to excited). To examine emotions in experimental settings, the International Affective Picture system (IAPS) [24] provides a set of colored pictures which are rated along those two dimensions. However, both dimensions are not independent of each other, since highly pleasant and unpleasant pictures score in many cases also high on the arousal scale (see figure 5). Bradley et al. [25] described a method to examine the influence of valence while controlling for the effects of arousal in an immediate free recall and 1-year-delayed free recall task. In the immediate recall task, they found that highly arousing IAPS slides were better recalled than low arousing pictures. With regard to the valence scale, pleasant slides were remembered slightly better than unpleasant slides. The advantage for highly arousing material still persisted when recalled one year after the initial learning phase, whereas the slight recall advantage for pleasant material was no longer emergent.

Independent of subsequent recall, emotional pictures as those taken from the IAPS [24] have been shown to modulate the P300 component [26], or late positive potential (LPP) [27,28] and the positive slow wave (PSW) [29]. Those components are more positive going for pleasant and unpleasant compared to neutral pictures. Some research found a stronger positivity for negative IAPS pictures, [28]. Dolcos and Cabeza [30] reported that the emotion effect on parietal scalp regions is more sensitive to arousal, whereas it is more sensitive to valence on frontocentral electrodes. The neural origin of the emotion effect is not entirely known, but it is assumed that the arousal effect is generated by interactions between the amygdala and cortical regions, whereas valence-related activity reflects prefrontal cortex involvement [30].

We see at least three different ways in which affective stimuli might contribute to increased recall performance. First, affective stimulus material might elicit physiological and evaluative processes that are not evoked by non-emotional stimuli. Second, affective stimuli capture attention, which might lead to speeded processing and/or increased rehearsal [31]. For example, subjects are faster and more accurate in detecting emotional stimuli compared to neutral stimuli [32]. Third, there is a general recall advantage for items belonging to a common category compared to uncategorized items. One might argue that emotional items are semantically coherent [33], that is they are more similar to each other than non-emotional items. Taken together, those additional attributes may increase the distinctiveness or add additional retrieval cues during encoding and, thus, support later recall.

How non-integral emotional information affects memory processes has been the subject of recent research. A word can be made emotionally distinct in a non-integral manner by presenting a highly emotional but task-irrelevant picture prior to the neutral item [17] or in the background of the neutral word (this study). Smith, Dolan and Rugg [34] examined ERPs elicited by objects in an old-new-recognition paradigm. Prior to the recognition test, the objects had been associated with pleasant, neutral or unpleasant IAPS background pictures. During the recognition phase correctly recognized items that had been encoded in emotional context differed from those learned in a neutral context around 800 ms. Encoding processes for non-integral emotional information was examined in a fMRI study conducted by Erk and colleagues [17]. They used IAPS pictures to induce positive or negative emotions prior to the presentation of the word. Recall performance was better for words preceded by positive but not negative pictures. Successful recall for positive encoding trials was predicted by activation of the right anterior parahippocampal and fusiform gyrus, whereas recalled items from negative encoding conditions were associated with amygdala activation. This supports the view that different pathways are involved when a person encodes words in a neutral, positive or negative context.

### The current study

The study presented here uses a von Restorff paradigm comprising the presentation of word lists, in which one word was made distinct by either changing an integral attribute (font color of one of the words) or by changing a non-integral attribute (arousing, unpleasant instead of low arousing background pictures). For the sake of simplicity, the arousing and unpleasant (low valence) background condition will be referred to as "arousing background condition".) The following questions are addressed: First, it is assumed that there is an increased memory performance combined with an increased ERP positivity (P300-recall-relationship) when changing an integral stimulus attribute. In contrast to earlier studies [22,23], the changed integral item was font color rather than font size and subjects were not instructed to use a specific encoding strategy. Second, it is predicted that a P300-Recall-relationship might also be seen for words isolated by a task-irrelevant, but highly arousing background picture. In contrast to changes of an emotionally neutral, non-integral item attribute [23], emotionally arousing background information should facilitate rehearsal processes in working memory and result in an even stronger P300-recall relationship.

## Results

### Behavioral data

#### Free recall performance

**Figure 1:**
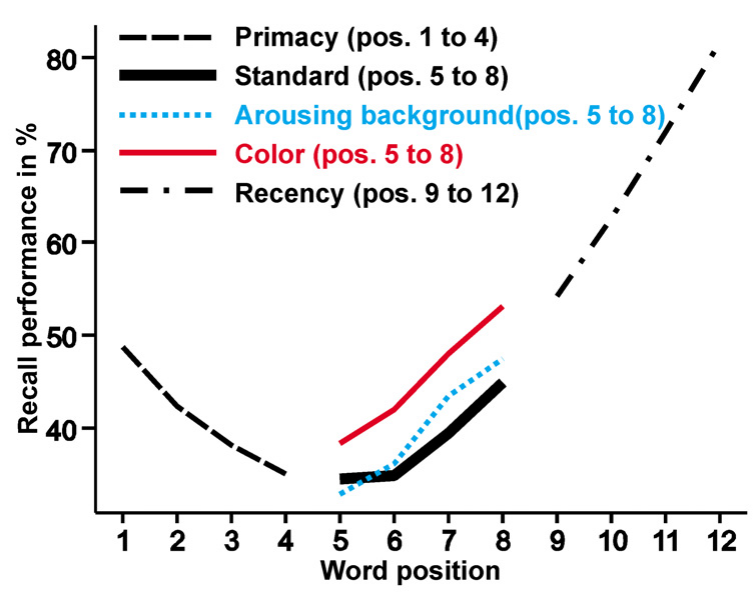
Serial Position Curves: Recall performance as a function of word position in list.

Overall, participants recalled 46.5 percent of the words. Words at the beginning of the list (position 1 to 4, primacy effect) were recalled in 41.1 percent, at the end of the list (position 9 to 12, recency effect) in 67.7 percent of the cases (Fig. 1). The Primacy and Recency effect will not be further discussed in this paper. In the following we will consider only trials that occurred at positions 5 to 8 of each list.

There was better recall performance for the *Color *condition compared to *Arousing background *and *Standard *conditions (45.3 vs. 39.9 vs. 38.4 % recall; F(2,34) = 5.78, P < .007; see Fig. 1 and Table 1). Paired t-tests revealed significant differences between *Standard *and *Color *(T (17) = -3.39; P < .003), *Color *and *Arousing Background *(T (17) = -2.43; P < .026), but no differences between *Standard *and *Arousing Background *(T (17) = -.71).

### ERPs

#### Condition differences

**Figure 2:**
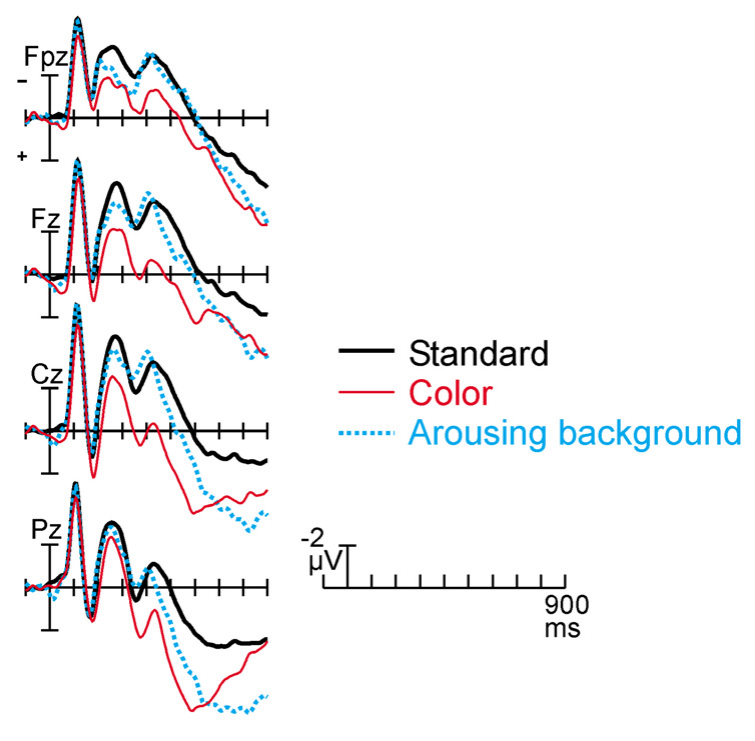
The P300 effect at midline electrodes. The figure depicts ERPs collapsed over recalled and non-recalled items.

Grand average ERPs are shown in Fig. 2 averaged across recalled and non-recalled words. The first clearly distinguishable component is the N100-P200 complex, which is typically seen for visually presented words.

*Color *isolates elicited a more positive ERP deflection around 180 ms, which was most pronounced at electrode-sites Fz and Cz, Fig. 2). A repeated measurement ANOVA with factors CONDITION (*Standard*, *Color*, *Arousing Background*) and SITE (Fpz, Fz, Cz, Pz) based on mean amplitude in the 180–250 ms time window revealed a significant main effect of CONDITION (F(2,34) = 6.69; p < .004). Single comparisons confirmed that the *Color word condition *differed from the *Neutral *and the *Arousing *condition within the 180–250 ms time window. Three further time intervals based on visual inspection of the grand average waveforms were assessed, 250 to 350 ms; 450 to 700 ms and 700 to 900 ms. In the 250 to 350 ms interval there was a significant effect of CONDITION (F(2,34) = 4.09; p < .03); individual comparisons revealed that the ERP for *Color *words was more positive compared to *Standard *words. However, amplitude differences between standard and *Arousing Background *words did not reach statistical significance. The same pattern was found in the 450 to 700 ms time window (CONDITION (F(2,34) = 5.02; p < .03; *Color *words more positive than *Standard *words).

Within the 700 to 900 ms time window, there was a significant effect of CONDITION (F(2,34) = 4.92; p < .015). Individual comparisons revealed amplitude differences between both classes of isolates and the standard words, whereas there are no significant differences between both types of isolates.

#### DM-effect

**Figure 3:**
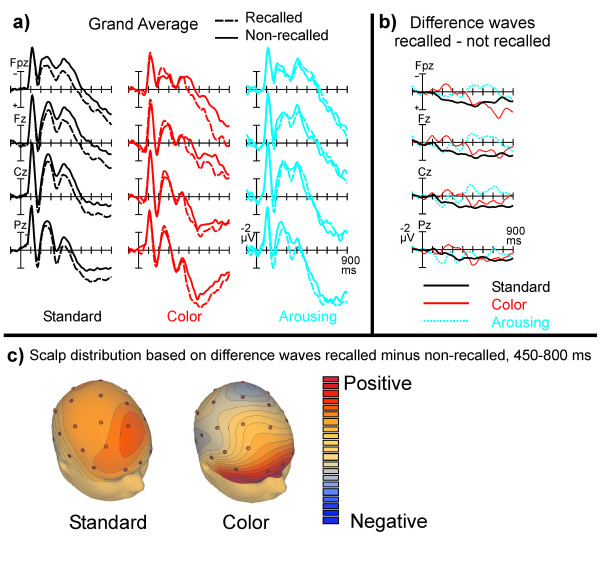
a) The DM-effect for midline electrodes Fpz, Fz, Cz and Pz. The left panel depicts grand average waveforms for the *Standard*, the *Color *and the *Arousing background *condition. b) displays difference waves (recalled minus non-recalled) for the three conditions. A clear DM-effect is only seen for the *Standard *and the *Color *word condition. c) Scalp distribution of the 450–800 ms time window.

Visual inspection of the data revealed a more positive going waveform for recalled compared to non-recalled items, starting at 250 ms for the *Standard *and at 450 ms for the *Color *condition. Importantly, this clear and long-lasting DM effect was not seen for the *Arousing Background *condition (see Fig. 3a and 3b).

Repeated measurement ANOVAs with factors RECALL (recalled, non-recalled) and SITE (Fpz, Fz, Cz, Pz) were conducted separately for each of the three conditions in the time windows 200 to 450 ms and 450 to 800 ms. In the 200 to 450 ms time window, there was a significant effect of RECALL in the *Standard *(F(1, 17) = 12.17, P < .003), but not in the *Color *(F(1,17) = .11; P < .74) or *Arousing Background *condition (F(1,17) = .93; P < .35). In the 450 to 800 ms time window, there was a significant main effect of RECALL for the *Standard *(F(1,17) = 13.17, P < .003) and the *Color *(F(1,17 = 5.17, P <.04), but not for the *Arousing Background *condition (F(1,17) = .40; P < .53). While visual inspection hinted at a small DM-effect in the *Arousing Background *condition between 300 and 400 ms an ANOVA on the mean amplitude in this time-window did not reveal a significant RECALL-effect (F(1,17) = 1.21; P < .29). Visual inspection might imply that there is an inverse DM-effect for the *Color *condition in the 200 to 300 ms time range. However, statistics conducted as described above revealed that the more negative waveform for recalled color isolates was not significant (F(1,17) = .87; P < .37).

The scalp distribution of the DM-effect in the 450–800 ms time window is depicted in Fig. 3c. For the *Standard *condition, the difference between recalled and non-recalled words have a frontoparietal distribution. In contrast, the DM-effect for the *Color *condition has a clear frontal maximum.

## Discussion

### Summary of results

Words that are isolated by a different color are remembered better than non-isolated words. This isolation advantage is not seen for words that are presented in front of a highly arousing picture. ERPs show that there is an increased P300 effect for words isolated by color and a delayed P300 for words isolated by a highly arousing background. An ERP modulation due to subsequent memory, a DM-effect, is seen for *Standard *and *Color *isolates, but not for *Arousing Background *words. This DM-effect starts later for colored words than for standard words.

### Behavioral data

The finding that words isolated by color are recalled better replicates earlier results [1,3,36]. However, there was no enhanced memory performance for words isolated by arousing background. This was unexpected, considering previous evidence that changing a non-integral item attribute results also in a VR-effect [23]. Consequently, the absence of the VR-effect in the *Arousing Background *condition can not merely be attributed to the non-integral nature of the isolation. Higher coherence among emotional stimuli [33] is also no major issue in the present study, since this would be expected to result in better than normal recall performance for the arousing background condition.

While it has been shown previously that emotionally enriched stimuli enhance memory performance [25,30,37], there is evidence that under some circumstances, memory performance can also be decreased in highly arousing situations. For example, Erk et al. [17] found no increased memory performance for words in negative compared to non-emotional encoding trials. They [17] pointed out that negative emotion might improve memory performance when it is part of the item to be encoded, but that it does not improve performance when it is part of the learning environment. Thus, to make a prediction about beneficial or detrimental effects of emotion during encoding, it is crucial to take the relationship between the arousing event and the stimulus to be encoded into consideration: Similar to the integral/non-integral distinction introduced above, the emotional content might be a central or a peripheral aspect of the encoding material [see [38] for overview]. This idea is derived from an old hypothesis introduced by Easterbrook in 1959 [39]: Arousal causes a narrowing of attention, which makes the aroused organism more sensitive to central information, but less sensitive to encoding material presented at the periphery. More recent research with human subjects supports this idea; central details were recalled more frequently than peripheral details in memories of shocking events [38,40]. Following this argumentation, the *Arousing background *condition in the present study can be regarded as a stimulus set consisting of an attention-capturing highly arousing, unpleasant element underlying an unemotional, less attention-attracting word. This puts the arousing background information rather than the word in the center of attention. This pattern is reversed in all other conditions, since the non-emotional background pictures do not act as an "attention-magnet" [38] and, thus, remains background information overlaid by a central, task-relevant word. That means that only in the *Arousing background *condition the background information could interact with memory processes. It is conceivable that the arousing information interrupted rehearsal, and, thus, decreases recall performance back to normal level.

Following this reasoning, one could argue that the lack of an VR-effect in the *Arousing background *condition and the stronger VR-effect in the *Color *condition is not caused by emotion, but merely by attention directed toward or away from the stimulus to be encoded. In line with this, additional attentional resources were allocated to the central color attribute in the *Color *condition, which results in increased recall performance. We do not think that this is a feasible alternative explanation, because other research [38,41] demonstrated a clear distinction between emotion-induced and attention-induced effects on memory performance: emotional contents increases memory selectively for central information, whereas attention-capturing, but unemotional stimuli do not selectively increase recall performance for central as well as peripheral information.

### Brain potentials

The most important ERP findings were a) a P300 effect, seen for the *Color *as well as *Arousing background *isolates, b) a DM-effect for the *Standard *and the *Color *condition, but not for the *Arousing background *condition, c) no positive slow wave in the *Arousing background *condition. These findings will now be discussed in detail.

The P300 for *Color *isolates as well as for *Arousing Background *isolates clearly indicates that subjects did process the arousing background information. Unlike previous research [23], which included also a non-integral, but non-emotional isolation attribute (frame around the word), the P300 in the *Arousing background *condition was not markedly reduced in amplitude compared to the integral isolation condition. This might reflect emotional processing which adds to the P300 amplitude elicited by pure non-integral properties. The large P300 for the *Arousing background *condition might be the neuropsychological correlate of the attention-capturing properties of emotional stimuli discussed above. The delayed peak latency for *Arousing backgrounds *might reflect increased stimulus complexity.

The DM-effect for the *Color Isolate *condition consists of a frontal and a parietal part: the latter likely represents a modulation of the P300, while the former is not easily explained in a P300 framework. The parietal DM-effect has often been discussed based on the context updating hypothesis developed to account for P300 modulations [3,20,22,23]. More intense context updating leads to a better memory trace, which increases the likelihood of successful recall. Words isolated by color contain additional information, which elicits more updating in working memory and leads to an improved memory trace during encoding and therefore to an increased VR-effect and P300-enhancement. As pointed out by Otten and colleagues [23], an integral part of the word (color in the current study, word size in [23]) cannot be separated from the words' informational content at an early processing stage; enhanced context updating might therefore be an obligatory processing step. The earlier onset of the DM-effect for the *Standard *compared to the *Color *condition might be due to the additional information content of the words isolated by color.

Otten and Donchin [23] pointed out "that some of the non-isolated words were distinctive in some sense, for semantic or contextual reasons. Specific words may appear to the subject to deviate from the general vocabulary used in the study. Or they may have personal associations that make some words distinct"(p.659). This might explain the DM-effect for non-isolated words (*Standard *words).

The generally increased P300 amplitude for *Arousing Background *stimuli was not associated with increased recall performance. Although unexpected and not supported by studies using IAPS pictures only [30], this finding is nevertheless in line with earlier observations [22,23] showing that the P300 amplitude is not necessarily a valid predictor for recall performance. Consequently, distinctiveness alone is not sufficient to elicit an enhanced P300 component for recalled items. Thus, having in mind that the *Arousing background *condition elicited a strong P300 independent of recall performance, we conclude that a P300-recall-relationship does not merely arise because a distinctiveness attribute triggers deeper and more extensive processing. It also matters how distinctiveness is achieved.

We see three ways to explain the missing P300-recall-relationship for the *Arousing Background *condition: First, the highly arousing pictures draw attention from the words toward the arousing background pictures. Unlike in the study conducted by Otten and Donchin [23], who concluded that the cognitive system could redirect the attentional resources away from a non-integral, but unemotional distinctiveness attribute, such an attentional redirection was not possible for our non-integral, but highly arousing isolation attribute. This resulted in an increased P300 for the *Arousing background *condition. It seems unlikely that recall performance for the words could benefit from the increased overall arousal level induced by the background picture, because arousing information increases memory performance for gist, but not for peripheral information [38]. Thus, the P300 is solely caused by the background pictures; there is no contribution of context updating elicited by the words. Consequently, there is also no P300-recall relationship for the recalled words. It appears that the recall-predicting part of the P300 can only be added by integral items. It is conceivable that a P300-recall-relationship would be seen if subjects were required to recall the content of the emotional background task. In this way, the emotional content would be an integral part *and *a gist of the encoding material. However, this assumption cannot be tested by the present study, because recall performance to the emotional background pictures was not acquired. Second, words with *Arousing Background *might be transferred into working memory, but are instantly overwritten by the arousing picture content. This would predict a memory performance for the *Arousing Background *condition that is even lower than for non-isolated words which was not the case. The third explanation is based on the earlier finding that subjects do not show a VR-effect in behavioral data and no P300-recall relationship in the ERPs when they were instructed to use an elaborative rather than a rote rehearsal strategy to encode word stimuli [21,22]. To some extent, this finding is similar to the results presented here. Since a P300-recall-relationship is not seen when subjects refrain from rote strategies, we assume that arousing context could interrupt the rehearsal strategy normally used in simple memory tasks.

In contrast to Donchin and Fabiani [22], the present experiment did not yield a positive slow wave for the *Arousing background *condition, which needs some further explanation. There are important differences between this putative "enforced strategy change" and the instructed strategy changes used elsewhere [22]: Instructing a subject to use a specific encoding strategy triggers top-down processes, which might influence memory performance and ERP components. There was no strategic instruction in the present study, the proposed strategy change was stimulus driven. These differences might contribute to the emergence of the recall sensitive frontal positive slow wave in Fabiani et al. [22].

On the other hand, a positive slow wave was also found when subjects used a rote encoding [23]. Otten and Donchin [23] attributed the positive slow wave to "the retrieval of preexisting knowledge about the word or to connecting the words with episodic information" (p. 658). Apparently, depending on task requirements and stimulus properties, successful encoding processes might be reflected in an increased P300 or an increased positive slow wave. We add another aspect, namely that recall performance and ERPs might be decoupled as seen in the *Arousing background *condition. It has to be kept in mind that fMRI revealed a number of deep brain structures who display strong DM-type effects. Especially the amygdala has been shown to be involved in processing of arousing information [17, 30, 43]. However, those structures are not directly accessible by ERP technique, and thus, their "DM-activity" can not be demonstrated by ERPs. Thus, the following conclusions have to be considered as tentative. As mentioned above, emotional information enhances recall performance for the gist, but not for peripheral information. Adolph and colleagues [43] linked this finding directly to amygdala functions: they found that patients with unilateral amygdala damage do not show enhanced memory for gist information when encoding in emotional situations. Thus, the amygdala, possibly in cooperation with the hippocampus, might be involved in recalling words from the emotional background condition.

A possible limitation of the current study is the fact that highly arousing but pleasant emotional background pictures (upper right quadrant of figure 5) were not used. Thus, future studies should examine, whether positive affective information might lead to a von Restorff effect in the ERP.

## Conclusion

The study presented here provides further evidence that under integral item presentation, the P300 is a valid, but not sufficient predictor of recall performance. Additionally, more frontally distributed processes should be considered in prediction of recall as well. To get a broader model of how information is transferred and maintained in memory, it is crucial to take factors like item integrity, emotional background information and attention-capturing stimulus properties into account, since these can abolish the DM-effect completely without affecting memory performance.

## Methods

### Subjects

Data were acquired from 23 right-handed female students from different faculties at the Otto-von Guericke-University Magdeburg (age range 20 to 35, mean age: 24, SD 3.9). All subjects were native speakers of German and had normal or corrected to normal vision. They received € 7 per hour after completion of each session. Data from 5 subjects had to be discarded due to excessive artifacts in the EEG-tracings. Thus, data of 18 subjects were analyzed. All participants gave written informed consent prior to the experimental session. The study protocol was approved by the ethics committee of Magdeburg University.

### Stimuli and procedure

#### Experimental setup

Each subject participated in 2 sessions, separated by at least one week and both consisting of a *Color *deviant and an *Arousing background *deviant block. The sequence of the blocks was counterbalanced across subjects and sessions.

All parameters except the distinctiveness manipulation were identical for the blocks: Each trial started with a fixation cross with a varying duration between 600 and 1000 ms, followed by a word with an IAPS picture background for 1500 ms. Pictures were presented with the same duration and onset as the words. All words were presented in capital letters on a black frame, which was situated on the central lower part of a colored background picture. The words covered between 5.1 and 7.4, and the pictures covered horizontally 8.5 degrees of visual angle. Twelve trials constituted a list. After each trial, subjects were prompted to verbally recall all words they remembered from the previous block. These answers were recorded with a computer microphone for later analysis. The subject initiated the next list by a button press. There were 40 lists per block, yielding 480 words per block, 960 words per session and 1920 words for the entire experiment (12 words × 40 lists × 2 blocks (arousing vs. color deviant) × 2 sessions). The same 480 words were used in all blocks, but the position of the words and the accompanying background pictures were allocated randomly. Thus, there was a new and unique picture-word sequence for every subject, block and session.

**Figure 4:**
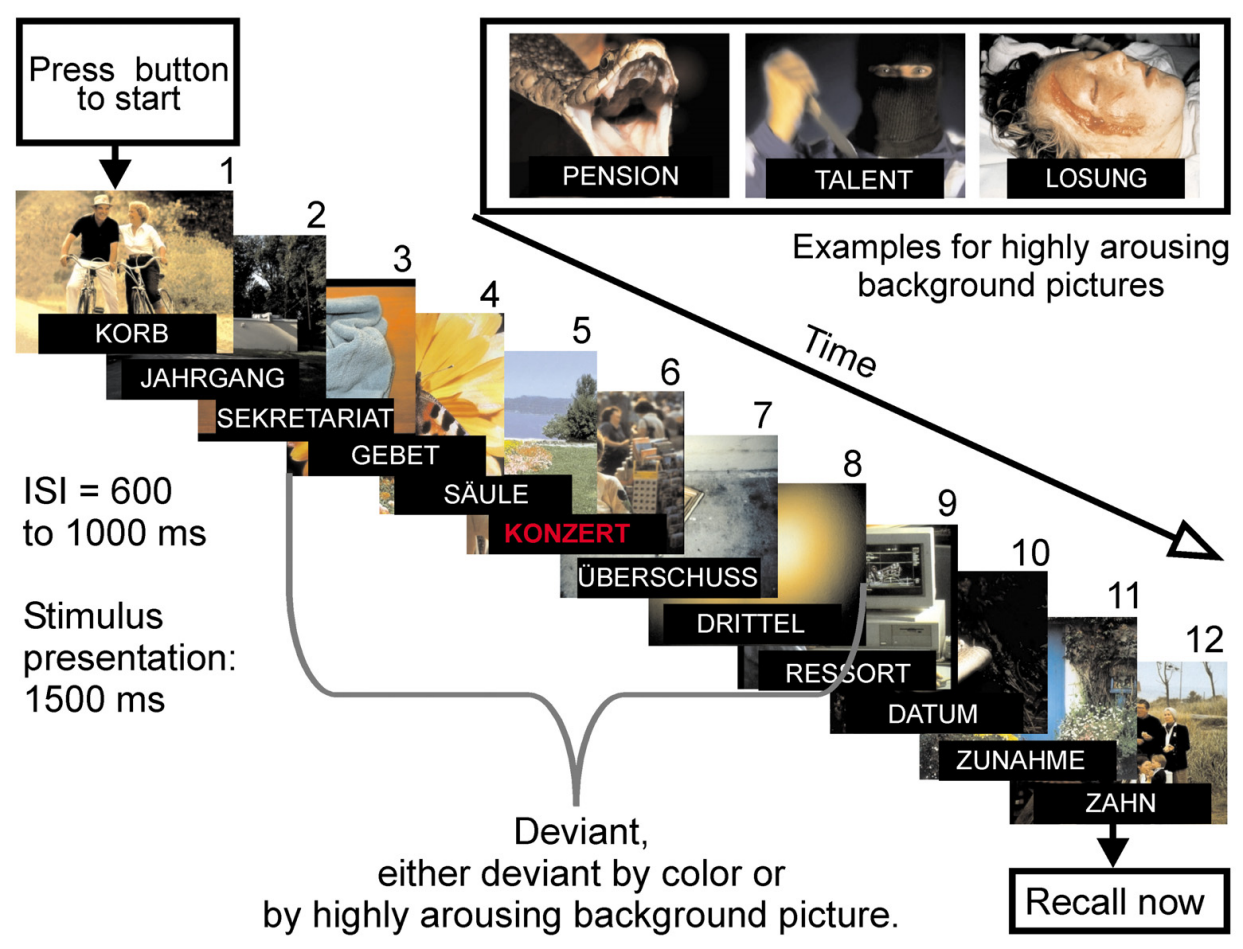
Experimental design. All words had colored background pictures, taken from the IAPS [24]. For the *Color *condition, one of the words on position 5 to 8 was colored (integral isolation). The IAPS background pictures in the *Color *condition were low on the arousal scale. In the *Arousing background *condition, all words had the same font color, but one of the background pictures on position 5 to 8 had a high emotional arousal value on the IAPS ratings and was unpleasant in valence (non-integral isolation). Words were aligned on the central lower part of the background picture.

Distinctiveness was created by changing either the word's color from white to colored (*Color deviant *condition, see below for color selection) or the word's background picture from low on the arousal scale to high on the arousing/low on the valence scale (unpleasant) (*Arousing background *condition, see below for picture selection). There was only one distinctiveness manipulation in each list, color isolate in the *Color *blocks, arousing background in the *Arousing background *blocks. Trials with a low arousing IAPS picture background (see below for picture selection) and white font words in the foreground constituted the *Standard *condition. To avoid confounds with the primacy and recency effect, the distinctiveness attribute was only changed on word positions from 5 to 8, and also behavioral and ERP data for the *Standard *condition were only generated from list position 5 to 8. The distinctiveness attribute appeared with the same frequency on each of the 4 middle positions. An overview of a single list is depicted in Fig. 4.

#### Word stimuli

480 German nouns were selected from the CELEX Lexical Database [44]. Four to 11 letter words were included and all words were emotionally neutral. Emotional neutrality of words was assured by a pre-study, in which 15 subjects were asked to mark every word they considered as not being emotionally neutral. This original list comprised 800 words. Words rated by more than 2 people to be not emotionally neutral were excluded. After completion of this procedure, 520 emotionally neutral words remained, 480 of which were randomly selected for the present study.

#### Background picture stimuli

**Figure 5:**
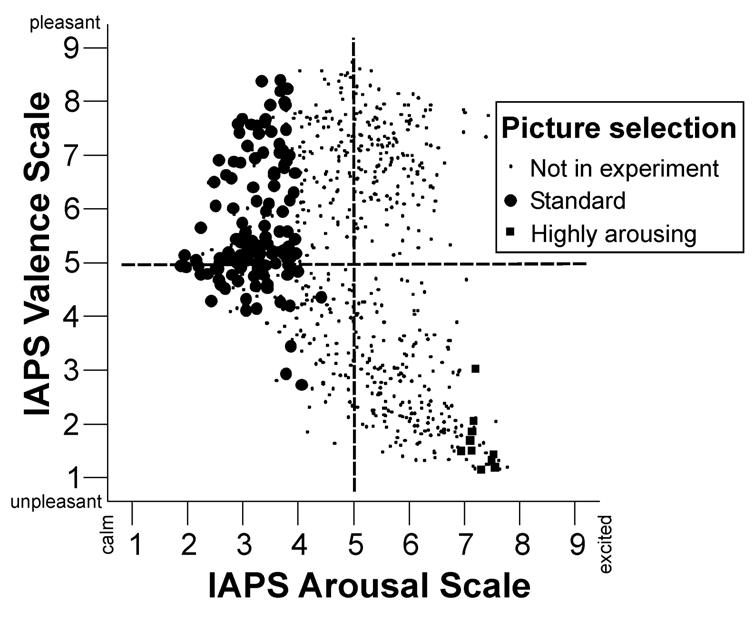
IAPS ratings on the IAPS arousal and valence scale for female subjects. Ratings are scored such that 9 represents a high rating on the dimension (high arousal, high pleasure), and 1 represents a low rating on each dimension (low arousal, low pleasure). Standard background pictures had a low arousal value; arousing background pictures had a high arousal value [24].

Words were presented in front of colored pictures taken from the International Affective Picture System (IAPS) [24]. The two primary dimensions of the IAPS are affective valence (ranging from unpleasant to pleasant) and arousal (ranging from calm to excited). Background pictures were selected based on their arousing values for female subjects provided with the IAPS manual. Low arousing pictures were taken as background in the *Color *deviant and the *Standard *condition, highly arousing pictures provided the background in the *Arousing background *condition. The arousal scale has 9 levels, with 1 depicting the lowest (calm), 5 a medium and 9 the highest arousal level (excitement). A picture was defined to be low arousing when the mean value of the arousing scale plus 0.5 SD did not exceed the value 5 on the IAPS arousal scale. Pictures with portrait alignment were excluded. Of the remaining pictures 120 were selected on a random basis (mean arousal value = 3.2; minimum = 1.9; max = 4.4) for the *Standard *condition (low arousing background). A picture was considered to be highly arousing and unpleasant when the mean arousal value minus 1 SD was above 5 and the valence was lower than 3.1. Extremely intrusive pictures were excluded. Ten pictures were chosen from the remaining set (mean arousal level 7.3; minimum 6.9; maximum 7.4). Pictures contained (e.g.) mutilations, dead bodies, snakes, and threatening situations. The following IAPS pictures were selected for the *Arousing background *condition: 1120, 3030, 3064, 3071, 3120, 3140, 3170, 6350, 6510, and 6540 (see Fig. 5).

Since the *Arousing Background *condition was made distinct by 10 different emotional backgrounds, 10 different colors from the 256 RGB color scale were selected for the color word condition. Only colors that are easy to discriminate were included.

#### Procedure

Subjects were seated in front of a monitor in an electrically shielded and sound attenuated cabin. They were instructed that they will be presented with pictures superimposed by words and that their task is to recall orally as many words as possible at the end of each 12-word-list. There was no instruction regarding the strategy for memorizing the words, and the distinctiveness manipulations were not explained to the subjects.

#### Data recording and analysis

During the encoding phase, the electroencephalogram (EEG) was recorded from 29 electrodes including all 19 standard locations of the 10/20 system with tin electrodes mounted in an elastic cap relative to a reference electrode placed on the left mastoid. Eye-movements were recorded with electrodes affixed to the right and left external canthi (horizontal electrooculogram (hEOG), bipolar recording) and at the left and right orbital ridges (vertical electrooculogram (vEOG), bipolar recording). Impedances of all electrodes were kept below 10 kΩ. Biosignals were amplified with a band-pass from 0.05 to 30 Hz and stored with a digitization rate of 250 Hz.

Prior to ERP data analysis, all trials containing artifacts were discarded, using a special purpose program with individualized peak-to-peak-amplitude criteria on vEOG, hEOG and head channels. Stimulus-locked ERPs were averaged for epochs of 1024 ms starting 100 ms prior to stimulus onset. Averages were computed for each condition (*Standard, Arousing background, Color*) separately for recalled and non-recalled words. Only trials positioned in middle word positions (positions 5 to 8) were included in ERP data analysis.

The mean number of trials included in each of the six conditions is listed in Table 2.

Although, after artifact correction, there were a few subjects with a low number of trials included in some of the averages (lowest trial number: 10), all averages provided clear signals.

## Authors' contributions

JR, DW and TFM designed the study. DW and SH acquired and analyzed the data. DW, JR and TFM drafted the manuscript.

## Figures and Tables

**Table 1:** Recall performance in percent for the *Standard*, the *Arousing Background *and the *Color *condition. Values are means across all 18 subjects, SDs are given in brackets. VRI indexes the Von-Restorff-index, computed by subtracting the recall percentage for *Standard *words from the emotional or color recall percentage.

	**Condition**		
	
	**Standard Pos 5–8**	**Arousing Background**	**Color**
**Recall**	38.4 (10.1)	39.9 (11.0)	45.3 (11.6)
**VRI**		1.5 (9.2)	6.9 (8.7)

**Table 2:** 

Standard recalled:	mean 127	SD = 44	range 80–236
Standard not recalled:	mean 197	SD = 54	range 81–309
Arousing recalled:	mean 22	SD = 6	range 14–34
Arousing not recalled:	mean 34	SD = 11	range 10–55
Color recalled:	mean 25	SD = 7	range 11–42
Color not recalled:	mean 32	SD = 12	range 15–56

## References

[B1] von Restorff H (1933). Ueber die Wirkung von Bereichsbildungen im Spurenfeld. Psychologische Forschung.

[B2] Cimbalo RS, Grunneberg MM, Morris PE, R.N. S (1978). Making something stand out: The isolation effect in memory performance.. Practical aspects of memory.

[B3] Fabiani M, Donchin E (1995). Encoding processes and memory organization: a model of the von Restorff effect. J Exp Psychol Learn Mem Cogn.

[B4] Cooper EH, Pantle AJ (1967). The Total-Time Hypothesis in Verbal Learning. Psychological Bulletin.

[B5] Hunt RR, Lamb CA (2001). What causes the isolation effect?. J Exp Psychol Learn Mem Cogn.

[B6] Ranganath C, Rainer G (2003). Neural mechanisms for detecting and remembering novel events. Nature Reviews Neuroscience.

[B7] Simons RF, Graham FK, Miles MA, Chen X (2001). On the relationship of P3a and the Novelty-P3. Biol Psychol.

[B8] Polich J, Kok A (1995). Cognitive and biological determinants of P300: an integrative review. Biol Psychol.

[B9] Strange BA, Henson RNA, Friston KJ, Dolan RJ (2000). Brain mechanisms for detecting perceptual, semantic, and emotional deviance. Neuroimage.

[B10] Strange BA, Dolan RJ (2001). Adaptive anterior hippocampal responses to oddball stimuli. Hippocampus.

[B11] Paller KA, Kutas M, Mayes AR (1987). Neural correlates of encoding in an incidental learning paradigm. Electroencephalogr Clin Neurophysiol.

[B12] Wagner AD, Koutstaal W, Schacter DL (1999). When encoding yields remembering: insights from event-related neuroimaging. Philos Trans R Soc Lond B Biol Sci.

[B13] Neville HJ, Kutas M, Chesney G, Schmidt AL (1986). Event-related brain potentials during initial encoding and recognition memory of congruous and incongruous words. Journal of Memory and Language.

[B14] Fernandez G, Weyerts H, Tendolkar I, Smid HG, Scholz M, Heinze HJ (1998). Event-related potentials of verbal encoding into episodic memory: dissociation between the effects of subsequent memory performance and distinctiveness. Psychophysiology.

[B15] Friedman D, Sutton S (1987). Event-related potentials during continuous recognition memory. Electroencephalogr Clin Neurophysiol Suppl.

[B16] Friedman D, Ritter W, Snodgrass JG (1996). ERPs during study as a function of subsequent direct and indirect memory testing in young and old adults. Brain Res Cogn Brain Res.

[B17] Erk S, Kiefer M, Grothe J, Wunderlich AP, Spitzer M, Walter H (2003). Emotional context modulates subsequent memory effect. Neuroimage.

[B18] Kirchhoff BA, Wagner AD, Maril A, Stern CE (2000). Prefrontal-temporal circuitry for episodic encoding and subsequent memory. J Neurosci.

[B19] Lisman JE, Grace AA (2005). The hippocampal-VTA loop: controlling the entry of information into long-term memory. Neuron.

[B20] Donchin E, Coles MG (1988). Is the P300 component a manifestation of context updating?. Behavior and Brain Sciences.

[B21] Karis D, Fabiani M, Donchin E (1984). "P300" and memory: Individual differences in the von Restorff effect. Cognitive Psychology.

[B22] Fabiani M, Karis D, Donchin E (1990). Effects of mnemonic strategy manipulation in a Von Restorff paradigm. Electroencephalogr Clin Neurophysiol.

[B23] Otten LJ, Donchin E (2000). Relationship between P300 amplitude and subsequent recall for distinctive events: dependence on type of distinctiveness attribute. Psychophysiology.

[B24] Lang P, Bradley M, Cuthbert B (1999). International affective picture system (IAPS): instruction manual and affective ratings. Technical Report A-4, The Center for Research in Psychophysiology.

[B25] Bradley MM, Greenwald MK, Petry MC, Lang PJ (1992). Remembering pictures: pleasure and arousal in memory. J Exp Psychol Learn Mem Cogn.

[B26] Keil A, Bradley MM, Hauk O, Rockstroh B, Elbert T, Lang PJ (2002). Large-scale neural correlates of affective picture processing. Psychophysiology.

[B27] Schupp HT, Cuthbert BN, Bradley MM, Cacioppo JT, Ito T, Lang PJ (2000). Affective picture processing: the late positive potential is modulated by motivational relevance. Psychophysiology.

[B28] Ito TA, Larsen JT, Smith NK, Cacioppo JT (1998). Negative information weighs more heavily on the brain: the negativity bias in evaluative categorizations. Journal of Personality and Social Psychology.

[B29] Amrhein C, Muhlberger A, Pauli P, Wiedemann G (2004). Modulation of event-related brain potentials during affective picture processing: a complement to startle reflex and skin conductance response?. Int J Psychophysiol.

[B30] Dolcos F, Cabeza R (2002). Event-related potentials of emotional memory: encoding pleasant, unpleasant, and neutral pictures. Cogn Affect Behav Neurosci.

[B31] Ochsner KN (2000). Are affective events richly recollected or simply familiar? The experience and process of recognizing feelings past. Journal of Experimental Psycholology: General.

[B32] Ohman A, Flykt A, Esteves F (2001). Emotion drives attention: detecting the snake in the grass. J Exp Psychol Gen.

[B33] Maratos EJ, Allan K, Rugg MD (2000). Recognition memory for emotionally negative and neutral words: an ERP study. Neuropsychologia.

[B34] Smith AP, Dolan RJ, Rugg MD (2004). Event-related potential correlates of the retrieval of emotional and nonemotional context. J Cogn Neurosci.

[B35] Wallace WP (1965). Review of the historical, empirical, and theoretical status of the von Restorff phenomenon. Psychological Bulletin.

[B36] Palomba D, Angrilli A, Mini A (1997). Visual evoked potentials, heart rate responses and memory to emotional pictorial stimuli. Int J Psychophysiol.

[B37] Reisberg D, Hertel P, Reisberg D, Heuer F (2004). Memory for Emotional Events. Memory and Emotions.

[B38] Easterbrook JA (1959). The effect of emotion on cue utilization and the organization of behavior. Psychological Review.

[B39] Berntsen D (2002). Tunnel memories for autobiographical events: Central details are remembered more frequently from shocking than from happy experiences. Memory and Cognition.

[B40] Christianson SA, Loftus EF (1991). Remembering emotional events: The fate of detailed information. Cognition and Emotion.

[B41] Adolphs R, Tranel D, Buchanan TW (2005). Amygdala damage impairs emotional memory for gist but not details of complex stimuli. Nat Neurosci.

[B42] Baayen RH, Piepenbrock R, Gulikers L (1995). The CELEX Lexical Database (Release 2) [CD-ROM].

